# Localized demodicosis associated with *Demodex canis* and *Demodex cornei* infestation in a dog from Mexico

**DOI:** 10.1590/S1984-29612026018

**Published:** 2026-06-15

**Authors:** Elsa Carolina Landeros Gálvez, Lucía Teresa Fuentes Guardiola, Jerónimo Landeros Flores, Oscar Ángel Sánchez Flores

**Affiliations:** 1 Universidad Autónoma Agraria Antonio Narro – UAAAN, Departamento de Parasitología Agrícola, Saltillo, Coahuila, México

**Keywords:** Canine demodicosis, Demodex, mixed infestation, Mexico, Demodicose canina, Demodex, infestação mista, Mexico

## Abstract

Canine demodicosis is a cutaneous parasitic disease caused by excessive proliferation of mites of the genus *Demodex*. This study reports a case of localized demodicosis associated with a concurrent infestation by *Demodex canis* and *Demodex cornei* in an adult mixed-breed female dog from the state of Coahuila, northern Mexico. The animal presented two focal alopecic lesions in the nasal and perioral regions, with no evidence of secondary infection. Deep skin scrapings were collected and examined by light microscopy. Species-level identification was based on diagnostic morphological characters, including opisthosomal shape and proportions, supracoxal spine morphology, and pharyngeal bulb structure, confirming the simultaneous presence of both species. This study represents the first confirmed record of *D. canis* and *D. cornei* in domestic dogs from the state of Coahuila and contributes to the knowledge of geographic distribution and diversity of demodecid mites in Mexico.

## Resumo

A demodicose canina é uma parasitose cutânea causada pela proliferação excessiva de ácaros do gênero *Demodex*. Neste estudo, relata-se um caso de demodicose localizada associada a uma infestação concomitante por *Demodex canis* e *Demodex cornei* em uma cadela sem raça definida, adulta, do estado de Coahuila, norte do México. O animal apresentou duas lesões alopécicas focais nas regiões nasal e perioral, sem evidência de infecção secundária. Foram realizados raspados cutâneos profundos, e o material obtido foi examinado por microscopia de luz. A identificação em nível de espécie baseou-se em caracteres morfológicos diagnósticos, incluindo a forma e as proporções do opistossoma, a morfologia dos espinhos supracoxais e a estrutura do bulbo faríngeo, confirmando-se a presença simultânea de ambas as espécies. Este trabalho constitui o primeiro registro confirmado de *D. canis e D. cornei* em cães domésticos no estado de Coahuila e contribui para o conhecimento da distribuição geográfica e da diversidade de ácaros demodécidos no Mexico.

## apagar

The family Demodicidae (Acari: Trombidiformes) comprises highly specialized ectoparasitic mites that inhabit the hair follicles and sebaceous glands of mammals (Mullen & O’Connor, 2019). Within this family, the genus *Demodex* includes numerous host-specific species parasitizing a wide range of mammalian hosts. In domestic dogs, three species of *Demodex* have been recognized, of which *Demodex canis* is the most important and best-known species due to its frequent association with clinical demodicosis (Saari et al., 2019). The remaining species, *Demodex injai* and *Demodex cornei*, can be distinguished from *D. canis* primarily by differences in body size and diagnostic morphological features. *Demodex injai* is substantially larger than *D. canis*, with adult males and females reported to be approximately 100% and 50% larger, respectively, and has been demonstrated to be genetically distinct (Sastre et al., 2012). In contrast *D. cornei* is markedly shorter than *D. canis* and exhibits distinct taxonomic features, including differences in the morphology of the female opisthosomal organ, as well as diagnostic variation in the supracoxal spines, the structure of the pharyngeal bulbs bearing the subgnathosomal setae, and the claws on the leg tarsi (Izdebska & Rolbiecki, 2018), allowing it to be reliably separated from the other two species.

Although *Demodex* mites form part of the normal cutaneous microfauna, excessive proliferation can lead to a clinically significant condition known as demodicosis, which manifests as demodectic mange (Mullen & O’Connor, 2019). Clinically, canine demodicosis is characterized by dermatological alterations such as alopecia, erythema, crusts and seborrhea, and the disease is commonly complicated by secondary bacterial infections (Sivajothi et al., 2015). Based on lesion distribution, demodicosis is commonly classified into localized and generalized forms. Localized demodicosis generally presents as one to four small well demarcated, partially alopecic macules or plaques, commonly affecting the facial region, particularly the periocular area and lateral commissures of the mouth; in contrast, generalized demodicosis involves more extensive and widespread lesions affecting multiple body regions (Saari et al., 2019). Demodicosis can also be further subdivided into juvenile-onset and adult-onset presentations; juvenile-onset demodicosis typically occurs before 18 months of age and is associated with immunological immaturity or genetic predisposition, whereas adult onset demodicosis occurs at four years of age or older and is frequently linked to underlying conditions causing immunosuppression (Foley et al., 2021).

Despite the widespread occurrence of canine demodicosis worldwide, the geographic distribution of *Demodex* species in dogs remains unevenly documented, particularly in Latin America. In Mexico, published records of canine *Demodex* are notably scarce. According to distributional compilations and checklists of ectoparasites of canids and felids in the country (Light et al., 2019), *Demodex canis* has been confirmed from domestic dogs in only two Mexican states, underscoring a pronounced geographic knowledge gap. Consequently, large regions of the country, especially northern Mexico, remain unrepresented despite substantial canine populations and diverse environmental conditions that may influence parasite occurrence. The documentation of a new locality record is therefore essential to refine national parasite inventories, validate existing checklists, and provide baseline data for epidemiological and clinical studies. In this context, the present study reports the first confirmed record of *Demodex canis* in domestic dogs from the state of Coahuila, northern Mexico, expanding its known distribution and contributing novel parasitological evidence to the understanding of canine demodicosis in the country.

**Figure 1:**
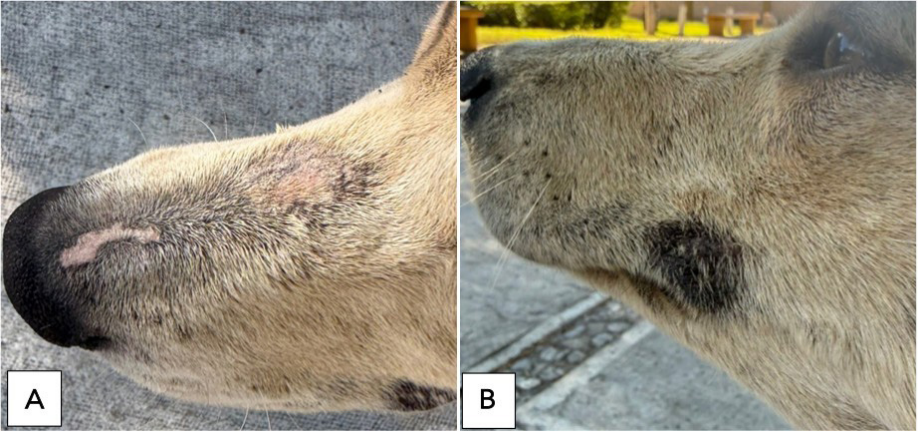
Localized demodicosis in a mixed-breed stray female dog. (A) Alopecic lesion on the nasale bridge with irregular margins and scaling. (B) Well demarcated, alopecic perioral lesion with marked hyperpigmentation. Original photographs by the first author.

A dermatological sample was obtained from an adult mixed-breed stray female dog, estimated to be 3-4 years of age, examined under field conditions in the southern outskirts of Saltillo, Coahuila, Mexico. At the time of examination, the animal exhibited a mildly reduced body condition score; however, no clinical evidence of severe malnutrition, dehydration or other underlying conditions were observed. The dog was alert, responsive, and displayed normal behavior. Dermatological examination revealed cutaneous alterations consistent with localized demodicosis. Two alopecic lesions were identified: one located on the nasal region and the other on the left side of the upper perioral area. Both lesions were approximately circular in shape and measured 1.5-2.0 cm in diameter. The nasal lesion appeared as a focal, irregularly shaped alopecic area located on the dorsal aspect of the nasal bridge, characterized by poorly defined margins, mild surface elevation, and evident scaling (Figure 1A). The affected skin exhibited a pinkish erythematous coloration, contrasting with the surrounding pigmented nasal planum. Hair shafts were sparse to absent within the lesion, and the surface texture appeared roughened compared with adjacent unaffected skin. In contrast, the perioral lesion presented as a well demarcated, focal area of uniform alopecia. The lesion exhibited a flat surface and smooth texture, lacking visible scaling, crusting, or surface irregularities, and showed marked hyperpigmentation compared with the surrounding normal integument (Figure 1B). The lesion margins were sharply defined, and the surface remained intact. Both lesions showed no evidence of ulceration, exudation, or secondary infection.

Deep skin scrapings were obtained from the affected areas using a sterile scalpel blade. Scraping was continued until mild capillary bleeding was observed to ensure adequate sampling of follicular contents. The collected material was temporarily mounted in immersion oil under a coverslip. The samples were examined under a Leica DME compound light microscope to confirm the presence of *Demodex* mites. Following microscopic confirmation of demodicosis, representative specimens were selected for permanent preparation. Individual mites were isolated using a glass capillary tube under a Zeiss inverted light microscope and subsequently mounted in Hoyer’s medium. The prepared slides were maintained at 45°C for approximately three to four weeks to allow complete clearing and hardening of the mounting medium. Morphological identification was subsequently performed under a compound light microscope based on established criteria for species-level differentiation within the genus *Demodex* (Izdebska & Rolbiecki, 2018).

**Figure 2:**
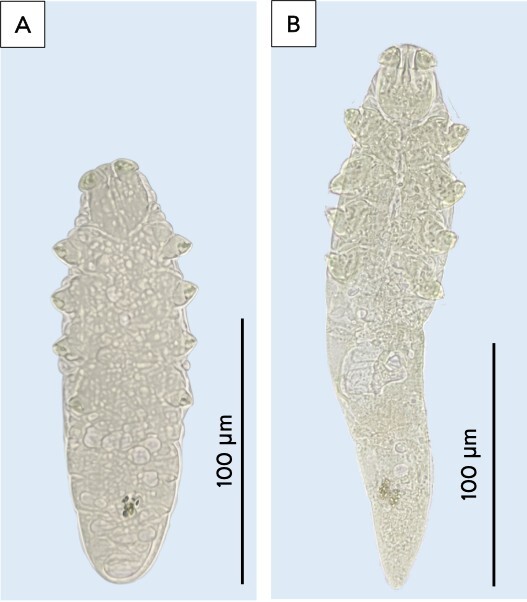
Adult females *Demodex* mites recovered from skin scrapings. (A) *Demodex cornei*, showing a short and compact body with a relatively short opisthosoma. (B) *Demodex canis*, characterized by an elongated body with a tapered opisthosoma. Scale bars = 100 µm. Original photographs by the first author.

Microscopic examination of skin scrapings revealed the presence of demodicid mites belonging to two distinct species of the genus *Demodex*. Based on diagnostic morphological characters, both *Demodex canis* and *Demodex cornei* were identified in the examined sample. Multiple adult specimens were observed, with females representing the predominant developmental stage. Two clearly differentiated morphotypes were recognized. One corresponded to *D. canis*, characterized by an elongated body form (Figure 2B), whereas the second morphotype was markedly shorter and more compact resulting in a stockier body appearance, consistent with *D. cornei* (Figure 2A). To avoid species determination based solely on overall body length, a more detailed morphological analysis was conducted. Species-level identification was supported by a comparative evaluation of multiple diagnostic morphological characters, which were fully consistent with the established taxonomic criteria described for each species.

**Figure 3:**
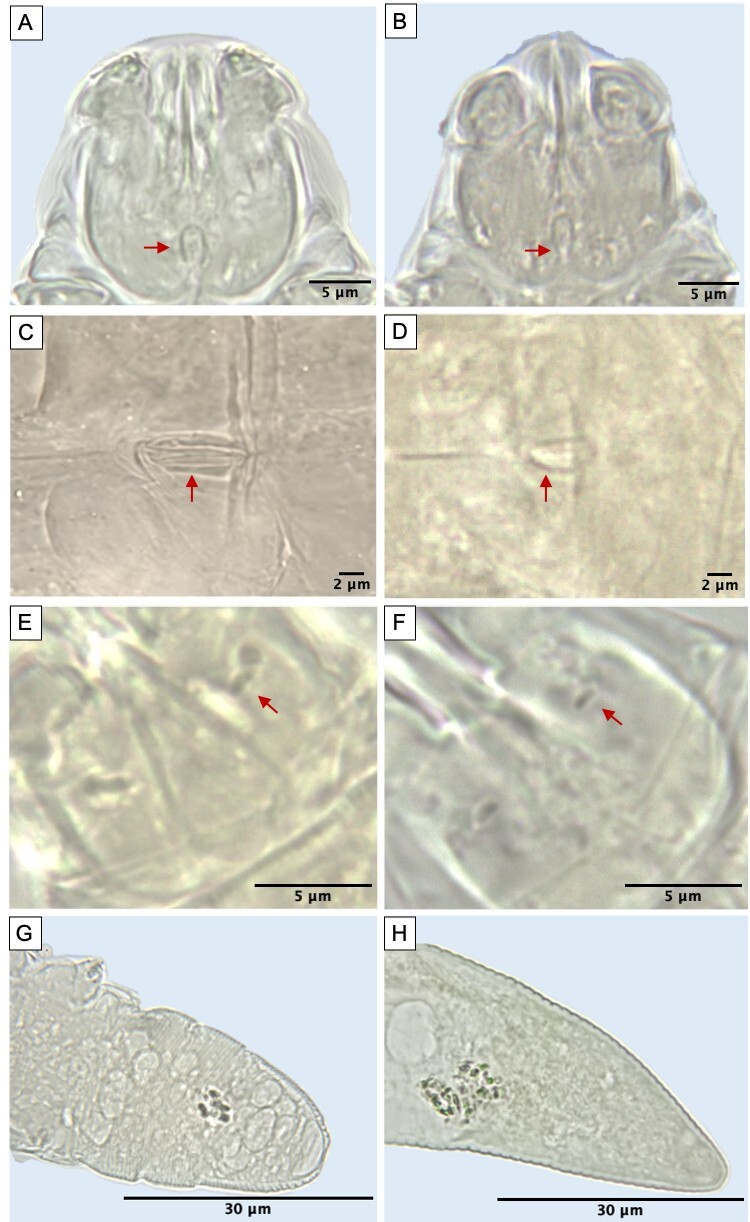
Key morphological characteristics of female specimens of *Demodex cornei* and *Demodex canis*. (A, C, E, G) *D. cornei*; (B, D, F, H) *D. canis*. (A-B) Pharyngeal bulb morphology: *D. cornei* showing an elongated, open bulb with a wide U-shaped outline, whereas *D. canis* exhibits a more compact bulb with strongly convex lateral margins and a narrower, more closed basal region. (C-D) Vulva: larger, broader in *D. cornei* and smaller, narrower in *D. canis*. (E-F) Supracoxal spines: foot-like, asymmetrical, and proximally expanded in *D. cornei*; peg-like, symmetrical, and shorter in *D. canis*. (G-H) Opisthosomal termination: rounded posterior end in *D. cornei* and blunt to sharply pointed posterior end in *D. canis*. Red arrows indicate the structures of interest.

Female specimens of *D. cornei* (n=2) possessed a short, test tube-like opisthosomal organ. The opisthosoma was oval and relatively short, measuring 71.85 ±13.5 µm in length, being shorter than the combined length of the gnathosoma and podosoma (86.0 ±0.6 µm), and terminated in a rounded posterior end (Figure 3G). The gnathosoma measured 20.8 ±2.0 µm in length and a basal width of 29.7 ±0.6 µm. The supracoxal spines were foot-like, asymmetrical, and proximally expanded, with a broad, flattened outline and an irregular terminal margin (Figure 3E). These spines were short and wide, measuring 3.7 ± 0.3 µm in length. The pharyngeal bulb was elongated and open, forming a wide, inverted U-shaped structure with parallel or slightly convergent lateral margins and a broad basal opening (Figure 3A) measuring 4.0 ±0.1 µm in length, with a basal width of 1.35 ±0.21 µm and a midpoint width of 1.9 ±0.1 µm. The vulva was comparatively broad (Figure 3C), measuring 7.1 ±0.0 µm in length and 3.0 ±0.2 µm in width.

In contrast, females of *D. canis* (n=3) exhibited an opisthosoma distinctly longer than the combined gnathosoma and podosoma, tapering posteriorly and ending in a blunt to sharply pointed tip (Figure 3H). The opisthosoma length (120.9 ± 8.9 µm) consistently exceeded the combined length of the gnathosoma and podosoma (89.2 ± 3.4 µm). The gnathosoma was trapezoidal with a length of 24.9 ± 2.8 µm, an anterior width of 16.1 ± 1.0 µm and a basal width of 34.7 ± 0.7 µm. The supracoxal spines were peg-like, symmetrical, and short (1.77 ± 0.23 µm) (Figure 3F). The pharyngeal bulb was compact, with strongly convex lateral margins and a narrowed, more closed basal region, resulting in a tighter inverted U-shaped outline (Figure 3B) measuring 4.4 ± 0.35 µm in length, 1.23 ± 0.15 at the base, and 2.5 ± 0.20 µm at the midpoint. The vulva was relatively small and narrow, measuring 5.3 ± 0.5 µm long and 2.1 ± 0.4 µm wide (Figure 3D).

The morphometric comparison of the examined female specimens suggested differences between *D. canis* and *D. cornei* that may support their differentiation at species-level (Table 1). However, these findings should be interpreted with caution, as the comparison was based on a limited number of individuals (n=3 for *D. canis* and n= 2 for *D. cornei*). In the specimens examined, females of *D. canis* were larger overall, with a greater total body length and, most notably, a substantially longer opisthosoma that consistently exceeded the combined length of the gnathosoma and podosoma. In contrast, females of *D. cornei* exhibited a shorter opisthosoma that was shorter than the sum of the anterior body regions, resulting in distinct proportional differences between both species. Additional diagnostic support was provided by the supracoxal spines, which were longer in *D. cornei* than in *D. canis*; however, beyond length alone, spine morphology represented an additional key diagnostic feature, with short, narrow tongue-like and symmetrical spines in *D. canis,* contrasted with broader, irregular, asymmetrical foot-shaped spines directed anteriorly in *D. cornei*. The pharyngeal bulb also differed subtly in shape and proportions, being more compact in *D. canis* and more elongated and open in *D. cornei*. Vulvar length and overall size were larger in *D. cornei*. Overall, the shape, size and position of the opisthosomal organ, together with differences in opisthosomal proportions, supracoxal spine morphology, vulvar dimensions, and pharyngeal bulb form, may represent useful complementary characters for the identification of female *Demodex* specimens, although their diagnostic consistency should be confirmed using a larger number of specimens.

**Table 1:** Comparative morphometric measurements (µm) of female *Demodex cornei* and *Demodex canis*. Values are expressed as mean ± standard deviation.

**Character**	***D. cornei* (n = 2)**	***D. canis* (n = 3)**
Body length	156 ± 9.9	209.7 ± 11.5
Gnathosoma length	20.8 ± 2.0	24.9 ± 2.8
Gnathosoma width (anterior)	18.4 ± 4.1	16.1 ± 1.0
Gnathosoma width (base)	29.7 ± 0.6	34.7 ± 0.7
Podosoma length	64.7 ± 3.0	67.0 ± 1.3
Podosoma width (medial point)	43.8 ± 1.8	45.8 ± 1.4
Gnathosoma + podosoma length	86.0 ± 0.6	89.2 ± 3.4
Opisthosoma length	71.85 ± 13.5	120.9 ± 8.9
Opisthosoma width (medial point)	38.8 ± 0.8	40.8 ± 0.6
Supracoxal spine length	3.7 ± 0.3	1.77 ± 0.23
Pharyngeal bulb length	4.0 ± 0.1	4.4 ± 0.35
Pharyngeal bulb width (base)	1.35 ± 0.21	1.23 ± 0.15
Pharyngeal bulb width (midpoint)	1.9 ± 0.1	2.5 ± 0.20
Vulva length	7.1 ± 0.0	5.3 ± 0.5
Vulva width	3.0 ± 0.2	2.1 ± 0.4

n = number of examined specimens (*D. cornei*, n = 2; *D. canis*, n = 3).

Measurements are given in micrometers (µm) and expressed as mean ± standard deviation (SD).

The simultaneous identification of *D. canis* and *D. cornei* in a single canine host underscores the importance of species-level identification in canine demodicosis. These findings add to previous observations suggesting that multiple *Demodex* species may coexist within the same host and remain undetected when diagnosis relies solely on clinical signs or superficial morphology. While *D. canis* and *D. cornei* have been associated with different cutaneous microhabitats (Moskvina, 2017; Thomson et al., 2023), their concurrent presence in the same host indicates that specific lesion patterns cannot be confidently attributed to a single species in this case. The mild and localized lesion observed in the present dog suggests that mixed infestations do not necessarily result in severe disease, as has been previously reported in some cases (Sivajothi et al., 2015; Yoon et al., 2020). However, this interpretation should be made with caution, since it is based on a single clinical case. Further studies specifically addressing mixed *Demodex* infestations and their associated clinical manifestations are required before more definitive conclusions can be drawn.

The distinction between *D. canis* and *D. cornei* in the present study was based on the combined assessment of several diagnostic morphological characters rather than on overall body size alone. This approach is particularly relevant given that short-bodied forms may be misinterpreted as errors in the specimens fixing and mounting or intraspecific variation of *D. canis*. The use of the morphology and setation of the gnathosoma, the localization and form of the opisthosomal organ, supracoxal spines, and pharyngeal bulb, were useful for specimen identification and highlight the practical applicability of the taxonomic framework proposed by Izdebska & Rolbiecki (2018). However, the absence of molecular data represents an important limitation of the present study, particularly in the context of the ongoing debate regarding the taxonomic status of *D. cornei*. Therefore, the morphological evidence presented here should not be interpreted as definitively resolving this controversy, but rather as providing additional morphological support for the distinction of these forms. Some studies have reported conspecificity based primarily on genetic similarity, suggesting that *D. canis, D. injai*, and *D. cornei* represent polymorphic forms of a single species (De Rojas et al., 2012), or interpreting *D. cornei* as a morphological variant of *D. canis* (Sastre et al., 2012). In this context, integrative approaches combining detailed morphological and molecular evidence will be necessary to clarify the taxonomic status of these mites more conclusively.

Finally, this record highlights the limited documentation of canine demodicid mites in Mexico, where species-level data remain scarce despite the frequent clinical diagnosis of demodicosis. The confirmation of both *D. canis* and *D. cornei* in northern Mexico helps narrow this knowledge gap and underscores the need for detailed morphological identification in future diagnostic and epidemiological studies. Overall, the present study shows that careful morphological analysis can detect previously unrecognized species diversity and suggests that mixed-species infestations may be more common than currently recognized.
